# Preoperative Red Cell Distribution Width and 30-day mortality in older patients undergoing non-cardiac surgery: a retrospective cohort observational study

**DOI:** 10.1038/s41598-018-24556-z

**Published:** 2018-04-18

**Authors:** H. R. Abdullah, Y. E. Sim, Y. T. Sim, A. L. Ang, Y. H. Chan, T. Richards, B. C. Ong

**Affiliations:** 1 Consultant, Department of Anaesthesiology, Singapore General Hospital, Singapore, Singapore Assistant Professor, Duke-NUS Medical School, Singapore, Singapore; 2 Senior Resident, Department of Anaesthesiology, Singapore General Hospital, Singapore, Singapore; 3 Medical Student, University of Tasmania School of Medicine, Hobart, Australia; 4 Senior Consultant, Department of Haematology, Singapore General Hospital, Singapore, Singapore; 5 Head, Biostatistics Unit, Yong Loo Lin School of Medicine, National University of Singapore, Singapore, Singapore; 6 Professor of Surgery, Division of Surgery, University College, London, United Kingdom; 7 Chairman Medical Board, Sengkang Health, Singapore, Singapore

## Abstract

Increased red cell distribution width (RDW) is associated with poorer outcomes in various patient populations. We investigated the association between preoperative RDW and anaemia on 30-day postoperative mortality among elderly patients undergoing non-cardiac surgery. Medical records of 24,579 patients aged 65 and older who underwent surgery under anaesthesia between 1 January 2012 and 31 October 2016 were retrospectively analysed. Patients who died within 30 days had higher median RDW (15.0%) than those who were alive (13.4%). Based on multivariate logistic regression, in our cohort of elderly patients undergoing non-cardiac surgery, moderate/severe preoperative anaemia (aOR 1.61, p = 0.04) and high preoperative RDW levels in the 3rd quartile (>13.4% and ≤14.3%) and 4th quartile (>14.3%) were significantly associated with increased odds of 30-day mortality - (aOR 2.12, p = 0.02) and (aOR 2.85, p = 0.001) respectively, after adjusting for the effects of transfusion, surgical severity, priority of surgery, and comorbidities. Patients with high RDW, defined as >15.7% (90th centile), and preoperative anaemia have higher odds of 30-day mortality compared to patients with anaemia and normal RDW. Thus, preoperative RDW independently increases risk of 30-day postoperative mortality, and future risk stratification strategies should include RDW as a factor.

## Introduction

Red cell distribution width (RDW) is an automated measure of the heterogeneity in erythrocyte sizes and is routinely performed as part of a Full Blood Count^1,2^. An increase in RDW, termed anisocytosis, reflects increased variation in the sizes of red blood cells (RBC) attributable to the presence of small and large RBCs, or both. Anisocytosis can be acquired, from nutritional deficiency such as iron (small RBC), vitamin B-12 and folate deficiency (large RBC), or in anaemia of chronic disease. RDW can also reflect underlying diseases such as in haemolytic anaemia and clinically significant thalassemic syndromes^3,4^. Traditionally, RDW has been used with other red cell indices (such as Mean Corpuscular Volume), to provide clues for underlying causes of anaemia, such as nutritional deficiencies and clinically significant thalassemia. More recently however, there is increasing interest in the role of RDW as a biomarker for inflammatory states and as a prognostication tool, with accumulating studies demonstrating increased RDW as an independent predictor for poorer outcomes among patients with ischaemic heart disease^5–8^, cardiac failure^9,10^, cerebrovascular disease^11,12^, cancer^13,14^ and patients who are critically ill in the Intensive Care setting^15^. More importantly, a number of studies have demonstrated a strong correlation between RDW and mortality in the older population^16–18^. Although the exact mechanisms remains unclear, given that higher RDW is associated with advancing age^3,10^ and higher disease burden^3,13^, RDW may serve as a novel biomarker that reflects multiple physiological impairments related to aging.

In the perioperative setting, increased RDW has also been found to be associated with long term outcomes such as one-year mortality^19,20^. Its association with shorter term outcomes such as 30-day mortality remains to be elucidated. With increasing proportion of elderly patients presenting for surgery, postoperative complications and patient outcomes are a major concern^21–23^. Consequently, there is a need to identify significant perioperative risk factors that allow accurate risk stratification for short term outcomes such as mortality and morbidity. This could facilitate meaningful informed patient consent and shared decision-making as well as facilitate targeted perioperative risk mitigation strategies^24,25^. Given that the RDW is routinely reported as a component of the Full Blood Count and is readily available for most patients undergoing surgery, understanding its prognostic potential could be very valuable and cost effective.

We aim to investigate the association between preoperative RDW and 30-day mortality among the elderly patients undergoing non-cardiac surgery and hypothesize that there is a significant association between increased RDW and 30-day mortality. Additionally, as anisocytosis is closely related to the presence of anaemia, we aim to explore the impact of anaemia on the relationship between RDW and 30-day postoperative mortality.

## Methods

### Data source

**Figure 1:**
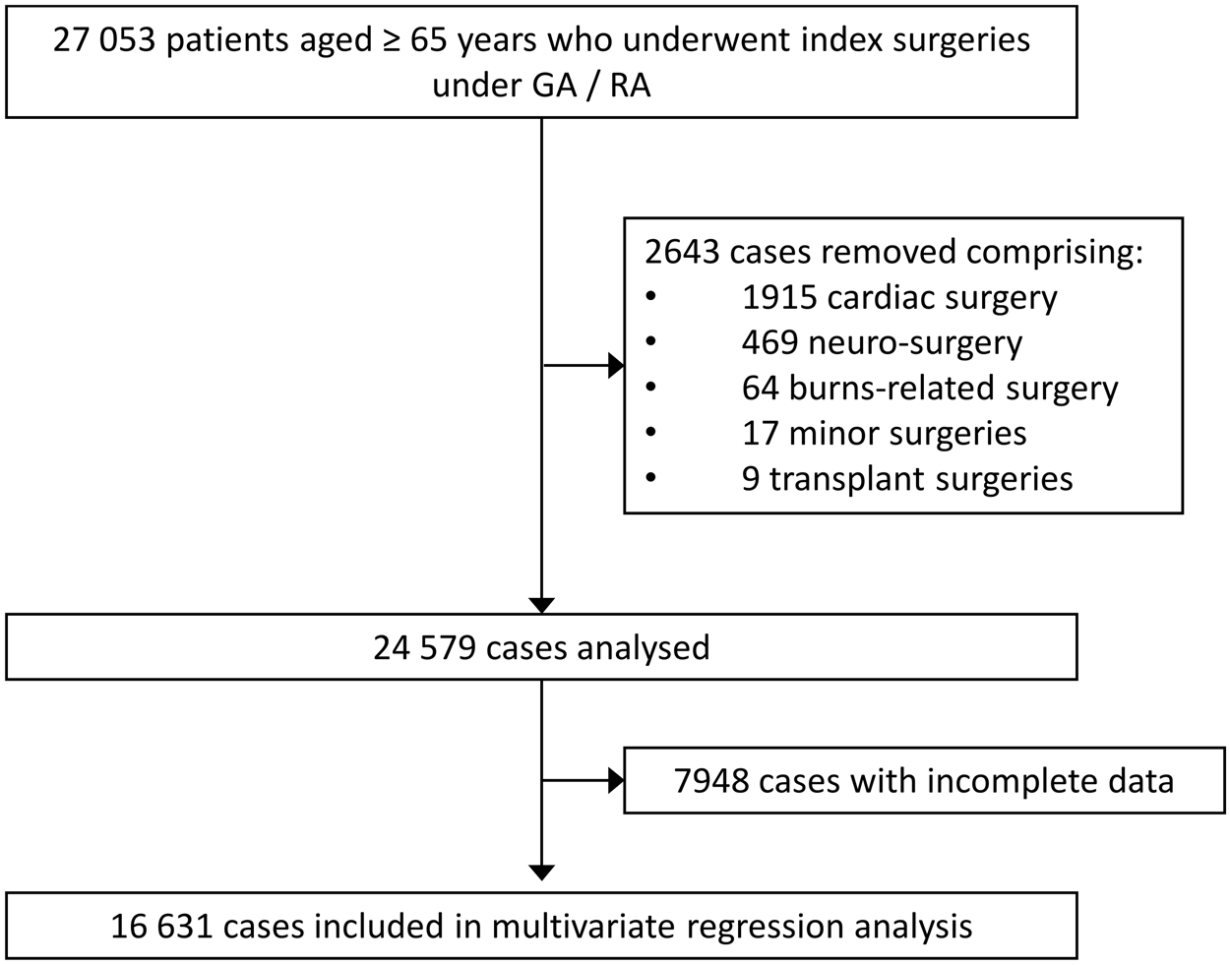
Study recruitment flow. Flowchart of Cohort Derivation. GA = general anesthesia; RA = regional anaesthesia.

We retrospectively analysed the electronic medical records of 27053 patients aged 65 and older who underwent surgery under general or regional anaesthesia between 1 January 2012 and 31 October 2016 in Singapore General Hospital, a 1700-bedded tertiary academic hospital in Singapore. Institutional Review Board approval was obtained (Singhealth CIRB 2014/651/D) prior to the start of the study and the study was performed in accordance with relevant guidelines and regulations. Due to the retrospective nature of the review, waiver of informed consent from the patient was approved by the CIRB. Clinical records were sourced from our institution’s clinical information system (Sunrise Clinical Manager (SCM), Allscripts, IL, USA) and stored in our enterprise data repository and analytics system (SingHealth-IHiS Electronic Health Intelligence System - eHINTS), which integrates information from multiple healthcare transactional systems including administration, clinical and ancillary systems. Mortality data on the system was synchronized with the national death registry records, ensuring a complete follow-up. We excluded patients who underwent cardiac surgery, neurosurgery, transplant and burns surgery due to their categorically higher blood transfusion requirement and mortality rate. Only the outcome of the index surgery was evaluated if a patient underwent multiple surgeries during the data collection period. Our final dataset comprised of 24579 patients (Fig. 1).

Data collected include patient demographics as well as preoperative comorbidities - ASA-PS score, previous cerebrovascular accidents (CVA), ischemic heart disease (IHD), congestive cardiac failure (CCF), diabetes mellitus (DM), Revised Cardiac Risk Index (RCRI) score^26^, chronic kidney disease and preoperative anaemia. Priority of surgery (emergency or elective) and surgical risk classification based on the 2014 ESC/ESA guidelines^27,28^ were recorded as well. Operative data included type of anaesthesia received, perioperative blood transfusion and need for reoperation within 30 days of the index surgery. There was incomplete data in the clinical database on conditions associated with raised RDW, such as iron deficiency, vitamin B12 or folate deficiency and myelodysplastic syndrome, hence these were not included in the analysis.

### Procedures and definitions

Full Blood Count were done in our institution’s College of American Pathologists accredited laboratory with Sysmex XN Automated Hematology Analyzer (Sysmex Corporation, Kobe, Japan) and Advia 2120i Hematology System (Siemens Healthcare Diagnostics Inc, USA). Preoperative laboratory results were taken as the latest blood results taken within 90 days before the surgery, and up to the day of surgery but before the start time of surgery. These results include the preoperative haemoglobin, red blood cell distribution width levels and serum creatinine levels. Anaemia was defined by the World Health Organisation (WHO)’s gender-based classification of anaemia severity^29^. Mild anaemia is defined as haemoglobin (Hb) 11–12.9 g/dL in males and 11–11.9 g/dL in females; moderate anaemia Hb 8–10.9 g/dL and severe anaemia Hb <8.0 g/dL. RDW is reported as a coefficient of variation (percentage) of red blood cell volume with the normal reference range for RDW in this hospital laboratory to be 10.9% to 15.7%.

Perioperative blood transfusion was defined as red blood cell (RBC) concentrate units given during the surgery and up to one month after the date of surgery. Pre-existing chronic kidney disease was defined based the estimated glomerular filtration rate (eGFR) that is derived from the latest preoperative serum creatinine levels by the MDRD equation according to KDIGO guidelines^30^. The individual components of RCRI were defined as per the original study by Lee *et al.*^26^.

### Statistical analysis

Statistical analysis was done in IBM SPSS Statistics v21.0. We compared the demographic and perioperative variables between patients who are alive or dead within 30-days of surgery, and across RDW quartiles. We checked continuous variables such as age for normality. For continuous, non-parametric variables, the Mann-Whitney U test was used to test for a significant difference in median values between 2 groups, and the Kruskal Wallis for a significant difference in median values across more than 2 groups. For categorical variables, Chi-square test was used to compare the percentages between the groups.

Multivariate logistic regression was performed to determine independent predictors for 30-day mortality adjusted for demographic, perioperative clinical risk factors, surgical factors as well as RDW quartiles. We checked for multi-collinearity between surgical risk, ASA score and RCRI with the Spearman correlation analysis. We plotted the AUC for RDW in predicting 30-day mortality in our patient cohort as well as the test statistics for RDW at regular cut-off levels – 13%, 14%, 15%, 16% and 17%. In addition, to explore the interaction between RDW and anaemia, we repeated the logistic regression for 30-day mortality with anaemia stratified by the presence of elevated RDW. We took a RDW cut-off of 15.7%, with values above it considered to be elevated. An RDW value of 15.7% corresponds to 90th centile of our population, and is also our laboratory cut-off for the upper limit of normal. We performed bootstrapping to assess the influence of missing data on the stability of the results. The p values of both results are very consistent, and the p-values that were significant on the multivariate analysis remained significant at p < 0.046.

## Results

### Patient demographics and characteristics

The baseline characteristics of our study cohort is presented in Table 1. 348 (1.42%) patients died within 30 days of their index surgery. The median age of those who survived up to 30-days after surgery was 72.0, and is significantly lower (P < 0.001) compared to those who died within 30-days (77.0 years). A significantly (p < 0.001) higher proportion of patients who underwent emergency surgery died within 30-days (5.2%) compared to 0.6% in those who underwent elective surgery. The incidence of mortality increased with increasing units of perioperative blood transfusion from 0.9% in those who did not receive transfusion, to 10.6% in those who received 2 or more units. Similarly, incidence of mortality increased progressively with increasing severity of anaemia, from 0.5% in the non-anaemics to 4.6% in those with moderate/severe anaemia. Moreover, the median RDW levels were also significantly higher in those who died (15.0) compared to those who were alive (13.4), P < 0.001.

**Table 1:** Characteristics of patients who died within 30-days compared to those alive after 30-days.

		Alive in 30 days (N = 24, 231)	Dead within 30 days (N = 348)	P-value
**Patient demographics**				
Age, median (IQR)		72.0 (68.0–77.0)	77.0 (72.0–82.8)	<0.001
Gender	Male	11471 (47.3%)	200 (57.5%)	<0.001
	Female	12760 (52.7%)	148 (42.5%)	
Race	Chinese	20695 (85.4%)	270 (77.6%)	<0.001
	Malay	1233 (5.1%)	39 (11.2%)	
	Indian	1103 (4.6%)	18 (5.2%)	
	Others	1200 (5.0%)	21 (6.0%)	
**Operation details**				
Priority of Operation	Elective	19919 (82.2%)	113 (32.5%)	<0.001
	Emergency	4312 (17.8%)	235 (67.5%)	
Surgical Risk	Low	9632 (39.8%)	96 (27.6%)	<0.001
	Moderate	13110 (54.1%)	195 (56.0%)	
	High	1489 (6.1%)	57 (16.4%)	
Type of Anaesthesia	GA	17084 (70.5%)	271 (77.9%)	0.003
	RA	7147 (29.5%)	77 (22.1%)	
Repeat Operation(s) within 30 days	Yes	1432 (5.9%)	64 (18.4%)	<0.001
	No	22799 (94.1%)	284 (81.6%)	
Perioperative Packed Red Blood Cell Transfusion	0 unit	22088 (91.2%)	206 (59.2%)	<0.001
	1 unit	1720 (7.1%)	92 (26.4%)	
	2 or more units	423 (1.7%)	50 (14.4%)	
**Patient characteristics**				
ASA-PS	1&2	16112 (69.0%)	43 (14.5%)	<0.001
	3	6816 (29.2%)	158 (53.2%)	
	4&5	436 (1.9%)	96 (32.3%)	
Preoperative Anaemia	None	14562 (61.1%)	66 (19.4%)	<0.001
	Mild	5097 (21.4%)	74 (21.7%)	
	Moderate/Severe	4190 (17.6%)	201 (58.9%)	
RCRI scores	0	11288 (62.9%)	33 (15.3%)	<0.001
	1	4778 (26.6%)	76 (35.2%)	
	2	1393 (7.8%)	69 (31.9%)	
	3	391 (2.2%)	29 (13.4%)	
	4	89 (0.5%)	8 (3.7%)	
	5	15 (0.1%)	1 (0.5%)	
	6	0	0	
Presence of Individual components of RCRI	Ischemic heart disease	2619 (15.1%)	93 (43.5%)	<0.001
	Previous Cerebrovascular accident	1023 (5.9%)	33 (15.3%)	<0.001
	Diabetes mellitus on insulin	831 (4.6%)	27 (12.3%)	<0.001
	Congestive cardiac failure	476 (2.6%)	24 (10.9%)	<0.001
	Elevated Creatinine >2 mg/dL	1030 (4.9%)	73 (28.4%)	<0.001
Stage of kidney disease based on eGFR (mL/min/1.73 m^2^)	None or 1	8532 (36.5%)	76 (22.4%)	<0.002
	2	9874 (42.3%)	69 (20.4%)	
	3	3481 (14.9%)	77 (22.7%)	
	4–5	1465 (6.3%)	117 (34.5%)	
RDW, median (IQR)		13.4 (12.8–14.3)	15.0 (13.8–17.0)	<0.001

Legend IQR = interquartile range; GA = general anaesthesia; RA = regional anaesthesia; ASA-PS = American Society of Anaesthesiologists Physical Status; RCRI = Revised Cardiac Risk Index; eGFR = estimated Glomerular Filtration Rate; RDW = Red Cell Distribution Width.

Percentages are calculated across the respective rows.

### RDW and 30-day mortality

A comparison of the cohort across the various RDW quartiles is shown in Table 2. There were disproportionately more patients in the 4^th^ RDW quartile with higher ASA PS (4&5), moderate/severe preoperative anaemia, received perioperative blood transfusion of 2 units or greater, higher RCRI scores (4&5), higher stages of chronic kidney disease (4&5) as well as those who died within 30 days after surgery.

**Table 2:** Characteristics of patients across RDW quartiles.

		1^st^ quartile RDW≤12.8% N = 6540	2^nd^ quartile RDW > 12.8%and ≤ 13.4% N = 6025	3^rd^ quartile RDW >13.4% and ≤14.3% N = 5286	4^th^ quartile RDW > 14.3% N = 5823	P-value
**Patient characteristics**						
Age, median (IQR)		71 (67,75)	72 (68,76)	72 (68,77)	73 (68,78)	<0.001
Gender	Male	3142 (27.8%)	2805 (24.8%)	2470 (21.8%)	2904 (25.7%)	0.001
	Female	3398 (27.5%)	3220 (26.1%)	2816 (22.8%)	2919 (23.6%)	
Race	Chinese	5834 (28.8%)	5270 (26.0%)	4417 (21.8%)	4728 (23.3%)	<0.001
	Malay	231 (18.7%)	244 (19.8%)	355 (28.8%)	403 (32.7%)	
	Indian	212 (19.5%)	241 (22.2%)	278 (25.6%)	356 (32.8%)	
	Others	263 (23.8%)	270 (24.4%)	236 (21.4%)	336 (30.4%)	
**Operation details**						
Priority of Operation	Elective	5503 (28.7%)	5061 (26.4%)	4353 (22.7%)	4270 (22.3%)	<0.001
	Emergency	1037 (23.1%)	964 (21.5%)	933 (20.8%)	1553 (34.6%)	
Surgical Risk	Low	2778 (30.0%)	2420 (26.1%)	2034 (22.0%)	2024 (21.9%)	<0.001
	Moderate	3530 (27.1%)	3318 (25.5%)	2945 (22.6%)	3212 (24.7%)	
	High	232 (16.4%)	287 (20.3%)	307 (21.7%)	587 (41.5%)	
Type of Anaesthesia	GA	4538 (27.4%)	4120 (24.9%)	3619 (21.9%)	4259 (25.8%)	<0.001
	RA	2002 (28.0%)	1905 (26.7%)	1667 (23.4%)	1564 (21.9%)	
Repeat Operations within 30 days	None	6197 (27.9%)	5718 (25.7%)	4962 (22.3%)	5359 (24.1%)	<0.001
	1 or more	343 (23.9%)	307 (21.3%)	324 (22.5%)	464 (32.3%)	
Perioperative Packed Red Blood Cell Transfusion	0 unit	6245 (29.1%)	5680 (26.5%)	4862 (22.7%)	4677 (21.8%)	<0.001
	1 unit	288 (16.6%)	320 (18.4%)	378 (21.8%)	751 (43.2%)	
	2 or more units	7 (1.5%)	25 (5.3%)	46 (9.7%)	395 (83.5%)	
**Patient characteristics**						
ASA-PS	1&2	5016 (32.3%)	4353 (28.0%)	3423 (22.0%)	2741 (17.6%)	<0.001
	3	1270 (18.8%)	1404 (20.8%)	1594 (23.6%)	2490 (36.8%)	
	4&5	41 (7.7%)	77 (14.5%)	101 (19.0%)	312 (58.8%)	
Preoperative Anaemia	None	5054 (35.5%)	4341 (30.5%)	3263 (22.9%)	1596 (11.2%)	<0.001
	Mild	1064 (21.0%)	1139 (22.5%)	1230 (24.3%)	1636 (32.3%)	
	Moderate/Severe	422 (9.7%)	545 (12.5%)	793 (18.2%)	2591 (59.5%)	
RCRI scores	0	3786 (34.7%)	3051 (28.0%)	2252 (20.7%)	1809 (16.6%)	<0.001
	1	1124 (24.4%)	1079 (23.4%)	1028 (22.3%)	1382 (30.0%)	
	2	238 (16.6%)	285 (19.9%)	331 (23.1%)	580 (40.4%)	
	3	67 (16.1%)	56 (13.5%)	92 (22.2%)	200 (48.2%)	
	4–5	12 (10.7%)	13 (11.6%)	24 (21.4%)	63 (56.2%)	
Presence of Individual components of RCRI	Ischemic heart disease	592 (22.4%)	591 (22.4%)	606 (22.9%)	854 (32.3%)	<0.001
	Previous Cerebrovascular accident	241 (23.3%)	245 (23.7%)	224 (21.7%)	324 (31.3%)	<0.001
	Diabetes mellitus on insulin	172 (20.6%)	176 (21.1%)	170 (20.4%)	315 (37.8%)	<0.001
	Congestive cardiac failure	69 (14.0%)	77 (15.6%)	107 (21.7%)	240 (48.7%)	<0.001
	Elevated Creatinine >2 mg/dL	118 (10.7%)	161 (14.6%)	252 (22.9%)	570 (51.8%)	<0.001
Stage of kidney disease based on eGFR (mL/min/1.73 m^2^)	None or 1	2649 (30.9%)	2179 (25.4%)	1774 (20.7%)	1964 (22.9%)	<0.001
	2	2968 (30.0%)	2765 (27.9%)	2218 (22.4%)	1944 (19.6%)	
	3	746 (21.1%)	840 (23.7%)	910 (25.7%)	1043 (29.5)	
	4–5	151 (9.6%)	220 (13.9%)	361 (22.9%)	847 (53.6%)	
30-day mortality	No	6520 (27.9%)	5990 (25.7%)	5212 (22.3%)	5613 (24.1%)	<0.001
	Yes	20 (5.9%)	35 (10.3%)	74 (21.8%)	210 (61.9%)	

Legend RDW = Red Cell Distribution Width; IQR = interquartile range; GA = general anaesthesia; RA = regional anaesthesia; ASA-PS = American Society of Anaesthesiologists Physical Status; RCRI = Revised Cardiac Risk Index; eGFR = estimated Glomerular Filtration Rate.

Percentages are calculated across the respective rows.

### Multivariate analysis

Based on multivariate logistic regression analysis, high preoperative RDW levels within 3^rd^ quartile (OR 2.12, 1.13–3.99, p = 0.02) or 4^th^ quartile (aOR 2.85, 1.57–5.17, p = 0.001) were independent risk factors for postoperative 30-day mortality in our cohort of elderly patients who underwent non-cardiac surgery. Other independent predictors of 30-day mortality were older age 76 years and above (aOR 2.41, 1.61–3.63, p < 0.001), Malay ethnicity (aOR 1.88, 1.17–3.02, p = 0.01) emergency surgery (aOR2.99, 2.15–4.14, p < 0.001), repeat surgeries within 30 days (aOR 2.29, 1.51–3.45, p < 0.001), perioperative blood transfusion of 1 unit (aOR 1.72, 1.15–2.58, p = 0.009) or 2 or more units (aOR 2.50, 1.51–4.14, p < 0.001), increasing ASA-PS score (3 or higher), presence of moderate/severe preoperative anaemia (aOR 1.61, 1.03–2.52, p = 0.04), history of ischaemic heart disease (aOR 1.70, 1.22–2.36, p = 0.002) and grade 4/5 chronic kidney disease (aOR 2.00, 1.29–3.10, p = 0.002). The results of univariate and multivariate analyses are shown in Table 3. Based on spearman correlation analysis, ASA and Surgical Risk category had high degree of correlation with a R of 0.742; ASA and RCRI had a weak correlation with R of 0.384; Surgical risk category and RCRI had negligible correlation with R of 0.079 (all P values <0.01). However, all 3 variables were included in our multivariate analysis as we feel that the degree of invasiveness and extent of the surgery is a very relevant clinical predictor.

**Table 3:** Table showing results of univariate and multivariate logistic regression of the factors that influence 30-day mortality in the elderly.

		N	Unadjusted OR (95% CI)	P value	Adjusted OR (95% CI)	P value
**Patient characteristics**						
Age	65 to 69 years	9201	REF		REF	
	70–75 years	7938	1.81 (1.30–2.50)	<0.001	1.25 (0.78–2.00)	0.348
	>=76 years	7440	4.10 (3.07–5.49)	<0.001	2.41 (1.61–3.63)	<0.001
Gender	Male	11671	REF		REF	
	Female	12908	0.67 (0.54–0.82)	<0.001	0.94 (0.69–1.27)	0.67
Race	Chinese	20965	REF		REF	
	Malay	1272	2.42 (1.73–3.41)	<0.001	1.88 (1.17–3.02)	0.01
	Indian	1121	1.25 (0.77–2.02)	0.36	1.26 (0.66–2.43)	0.48
	Others	1221	1.34 (0.86–2.10)	0.20	0.99 (0.45–2.20)	0.98
**Operation details**						
Priority of Operation	Elective	20032	REF		REF	
	Emergency	4547	9.61 (7.66–12.05)	<0.001	2.99 (2.15–4.14)	<0.001
Surgical Risk	Low	9728	REF		REF	
	Moderate	13305	1.49 (1.17–1.91)	0.001	1.87 (1.28–2.71)	0.001
	High	1546	3.84 (2.76–5.35)	<0.001	1.38 (0.81–2.37)	0.24
Type of Anaesthesia	GA	17355	REF		REF	
	RA	7224	0.68 (0.53–0.88)	0.003	0.72 (0.50–1.04)	0.08
Repeat Operations within 30 days		1496	3.59 (2.72–4.73)	<0.001	2.29 (1.51–3.45)	<0.001
Perioperative Packed Red Blood Cell Transfusion	0 unit	22294	REF		REF	
	1 unit	1812	5.74 (4.46–7.37)	<0.001	1.72 (1.15–2.58)	0.009
	2 or more units	473	12.67 (9.17–17.52)	<0.001	2.50 (1.51–4.14)	<0.001
**Patient characteristics**						
ASA-PS	1&2	16155	REF		REF	
	3	6974	8.69 (6.19–12.18)	<0.001	2.79 (1.76–4.43)	<0.001
	4&5	532	82.50 (56.87–119.68)	<0.001	10.07 (5.78–17.55)	<0.001
RDW	1^st^ quartile RDW≤12.8%	6540	REF		REF	
	2^nd^ quartile RDW>12.8% and≤13.4%	6025	1.91 (1.10–3.30)	0.02	1.31 (0.65–2.64)	0.45
	3^rd^ quartile RDW>13.4% and≤14.3%	5286	4.63 (2.28–7.60)	<0.001	2.12 (1.13–3.99)	0.02
	4^th^ quartile RDW>14.3%	5823	12.20 (7.70–19.32)	<0.001	2.85 (1.57–5.17)	0.001
Preoperative Anaemia	None	14628	REF		REF	
	Mild	5171	3.20 (2.30–4.47)	<0.001	1.44 (0.90–2.29)	0.13
	Moderate/Severe	4391	10.58 (8.00–14.01)	<0.001	1.61 (1.03–2.53)	0.04
Presence of individual components of RCRI	Ischemic heart disease	2712	4.32 (3.29–5.68)	<0.001	1.70 (1.22–2.36)	0.002
	Previous Cerebrovascular accident	1056	2.91 (1.99–4.23)	<0.001	1.19 (0.76–1.85)	0.45
	Diabetes mellitus on insulin	858	2.92 (1.94–4.40)	<0.001	0.82 (0.50–1.36)	0.44
	Congestive cardiac failure	500	4.58 (2.97–7.06)	<0.001	1.09 (0.66–1.81)	0.73
Stage of kidney disease based on eGFR (mL/min/1.73 m^2^)	None or 1	8608	REF		REF	
	2	9943	0.79 (0.57–1.09)	0.15	0.69 (0.44–1.09)	0.11
	3	3558	2.48 (1.80–3.42)	<0.001	1.07 (0.68–1.67)	0.78
	4–5	1582	8.97 (6.68–12.03)	<0.001	2.00 (1.29–3.10)	0.002

Legend ASA-PS = American Society of Anaesthesiologists Physical Status; eGFR = estimated Glomerular Filtration Rate; GA = general anaesthesia; IQR = interquartile range; OR = odds ratio; RDW = Red Cell Distribution Width; REF = reference; RA = regional anaesthesia; RCRI = Revised Cardiac Risk Index.

### Performance of RDW in predicting 30-day mortality

**Figure 2:**
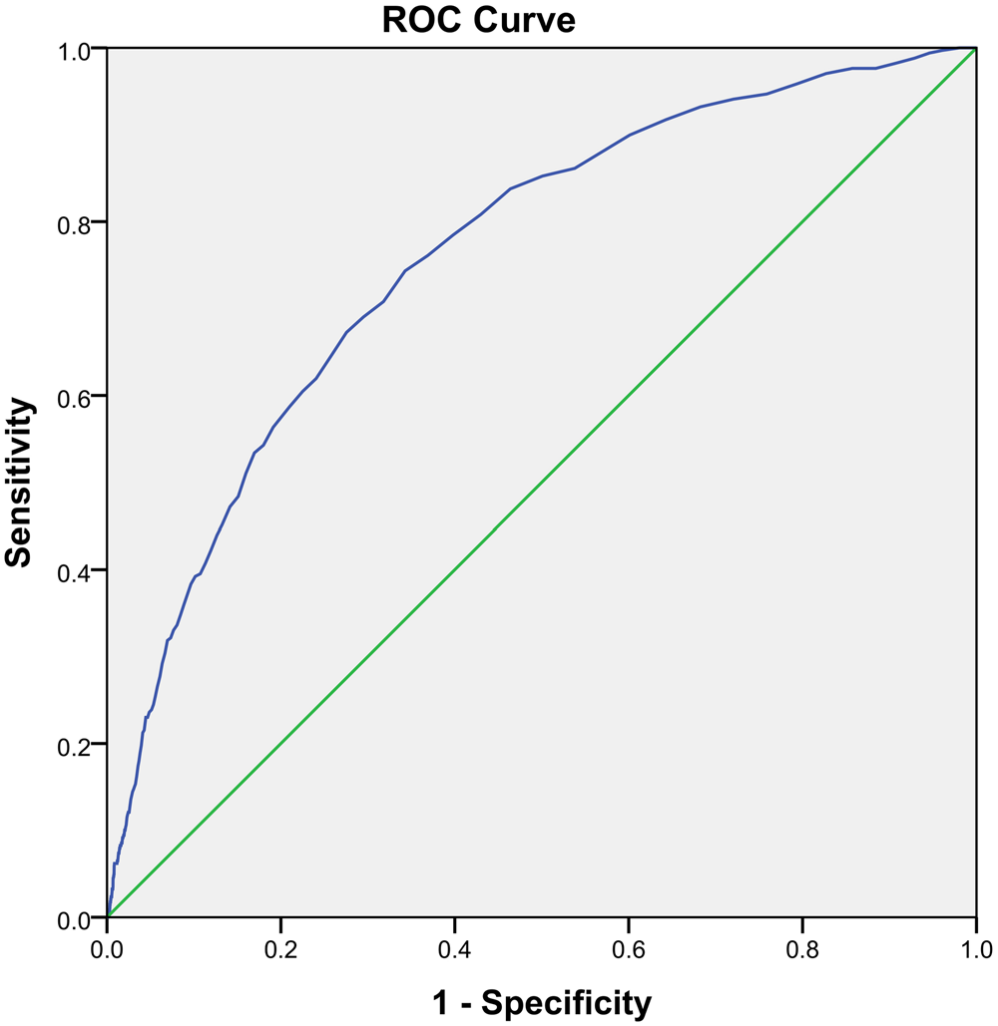
Receiver Operating Curve (ROC) of Red Cell Distribution Width (RDW) in predicting 30-day mortality.

**Figure 3:**
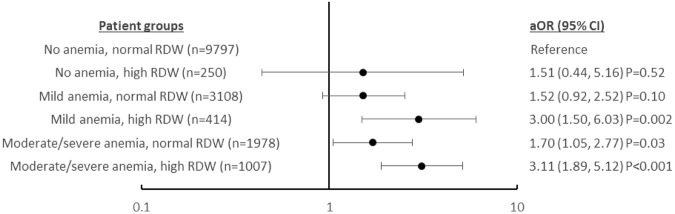
Adjusted Odds Ratio (aOR) for various degrees of anaemia stratified by red blood cell distribution width (RDW) levels and their 95% Confidence Intervals (CI) plotted on a log scale for the effect of anaemia stratified by RDW on 30-day mortality. Variables for adjustment are listed in Table 3, except anaemia and RDW.

As patients with each progressive RDW quartiles showed progressively increasing aOR of 30-day mortality compared to patients with RDW levels in the first quartile, we plotted the ROC for RDW in predicting 30-day mortality is shown as Figs 2 and 3. The area under the curve (AUC) was 0.761 (95% CI; 0.736–0.787). Furthermore, we calculated the sensitivity, specificity, and predictive values for 30-day mortality with incremental RDW values as shown in Table 4. The higher the RDW value, the lower its sensitivity and negative predictive value. However, specificity and positive predictive value increased with increasing RDW. Our chosen cut-off value of 15.7%, based on the upper limit of the range of normal population values validated in our laboratory, had a sensitivity of 39.5%, specificity of 89.3%, positive predictive value of 5.3% and negative predictive value of 99.0%.

**Table 4:** Test characteristics of Red Cell Distribution Width for 30-day mortality. Data presented as %.

RDW (centile)	Positive predictive value	Negative Predictive value	Sensitivity	Specificity
≥13 (35^th^)	93.2	31.7	1.9	99.7
≥14 (69^th^)	70.8	68.2	3.1	99.4
≥15 (84^th^)	51.0	84.0	4.4	99.2
≥16 (91^st^)	36.0	91.2	5.6	99.0
≥17 (94^th^)	25.4	94.5	6.2	98.9

RDW - Red cell distribution width.

### Incremental effect of RDW and Anaemia on 30-day mortality

As anaemia and higher RDW quartiles were independently associated with 30-day mortality in the multivariate regression analysis, we repeated the multivariate logistic regression by stratifying anaemia based on RDW levels (≤15.7% vs >15.7%) to examine for any interactions between anaemia and normal or high RDW. We found an incremental effect of high RDW (>15.7%) on anaemia in increasing the odds of 30-day mortality (Fig. 3). Strikingly, patients with mild anaemia and high RDW had almost twice the odds of 30-day mortality - aOR 3.00 (1.50–6.03) compared to patients with moderate/severe anaemia and normal RDW - aOR 1.70 (1.05–2.77), and similar odds of mortality as moderate/severe anaemia and high RDW - aOR 3.11 (1.89–5.12). This shows that high RDW has a consistent effect on mortality that is independent of anaemia. Unfortunately, we were unable to demonstrate the effect of high RDW in patients with no anaemia due to the small patient numbers (n = 250), which contributed to the wide confidence interval of the effect size -aOR 1.51 (0.44–5.16), p = 0.10.

## Discussion

In this study, we focused on older patients in the perioperative setting and demonstrated that a high RDW percentage is independently associated with 30-day all-cause postoperative mortality. This effect of high RDW on mortality is more pronounced in patients with anemia, and potentially could be used to improve the accuracy of clinical risk predictors. RDW is a simple parameter which reflects the degree of heterogeneity in erythrocyte volume and is routinely reported alongside other hematologic indices in the full blood count, which is a common blood investigation done prior to surgeries, and is thus available at no additional cost. Prior studies have demonstrated RDW to be a compelling independent risk factor for longer-term mortality in patients with cardiovascular diseases such as heart failure, acute myocardial infarction, coronary artery disease, pulmonary embolism, cardiac arrest, and stroke^11,31–38^. A meta-analysis revealed elevated RDW to be associated with aging and the progression of various types of disorders, and was also a strong independent predictor of long term survival in the older population^17^. Furthermore, multiple studies support the utilisation of RDW as a biomarker in risk stratification for mortality in general populations with or without cardiac diseases, cancer and other frequent comorbidities^5,18,20,31,32,39–41^. In the perioperative setting, elevated RDW has been associated with higher risks of one-year mortality after surgery^19,42,43^. Our study supports their finding by demonstrating that RDW also increases perioperative mortality in the short term (30-days). This suggests a role for RDW as a useful, cheap and convenient biomarker for predicting short term mortality in the perioperative setting.

While the exact biological mechanisms responsible for this association between increased RDW and mortality is currently unclear, previous studies suggested oxidative stress^44–46^, inflammation^44,47,48^, and malnutrition (such as iron, folate or vitamin B12 deficiency) as being the most plausible^3^. Oxidative stress, a common condition in most chronic human disorders, such as cancer, diabetes, heart diseases, liver failure and chronic kidney disease is associated with enhanced generation of reactive oxygen species and the consequent damage to nucleic acids, proteins and lipids has a profound influence on erythrocyte homeostasis and survival^49^. Inflammation, on the other hand, inhibits bone marrow function and iron metabolism^48^. Pro-inflammatory cytokines have also been determined to inhibit erythropoietin-induced erythrocyte maturation and proliferation and down regulate erythropoietin receptor expression, all of which increases RDW^50,51^. Hence, it is plausible that the stressful perioperative period may further increase the oxidative demands and inflammatory load beyond physiological tolerance, contributing to the poorer survival.

The presence of preoperative anaemia has been shown to be a strong predictor of postoperative mortality and morbidities^52–56^. Our findings add to current knowledge by demonstrating that high RDW (>15.7%) may further increase the odds ratio of 30-day mortality in those with anaemia, especially in patients with moderate/severe anaemia. This incremental effect suggests that the underlying reasons for increased RDW also have an impact on 30-day postoperative mortality. Admittedly, we were unable to show the impact of high RDW on mortality in patients with no anaemia, as we had few patients in our cohort with no anaemia and high RDW (n = 250), which may contribute to the wide confidence interval and statistical non-significance (aOR 1.51, 0.44–5.16), p = 0.102). Our choice of 15.7% as the cut-off for high versus normal RDW is in between the cut-offs of 14.5–16.0% chosen in other studies^10,57,58^ and was decided upon as it was the upper limit of normal population values validated in our laboratory. As shown in our cohort, as well as in other studies, mortality rate is higher in higher RDW quartiles^5,39,59^. In our study, despite having a study population of elderly patients who are at higher risk of adverse outcomes after surgery, the positive predictive value of the highest RDW cut-off of 17% is a mere 6.2%. Thus we feel at the choice of 15.7% is a reasonable trade-off between the desired sensitivity of a screening test for at-risk patients, yet balanced with a reasonable positive predictive value of 5.3%.

Apart from cost and the ability to standardize a test, an ideal biomarker for risk prediction in a population should include qualities such as the test’s independence from established risk factors and ability to extend predictability beyond current models and generalizability of the results^60^. Our study demonstrated that RDW was an independent risk factor for 30-day mortality in the multivariate analysis, however future studies can be done to investigate if the inclusion of RDW could improve the performance of currently available risk stratification models.

There is also a need for future studies to deepen the understanding of determinants for RDW, including identifying reversible factors such as nutritional deficiencies and oxidative stress, which may lead to improved outcomes after surgery. RDW could be a useful indicator of chronic health state and a practical addition to existing risk stratification strategy and shared decision making process.

### Limitations

The single-centre, retrospective observational design with short-term follow up are among the main limitations of this study. We also did not classify the causes of death and therefore could not examine the associations more closely. Furthermore, we did not have comprehensive data that may be associated with both RDW and surgical patient outcomes, such as presence of malignancy, underlying haematological disorders, nutritional deficiencies, socioeconomic status, and other known or unknown confounders. Therefore, we cannot exclude the possibility of residual confounding.

In the absence of a concrete understanding of the mechanism between increased RDW and mortality, it could be said that at this point, detecting elevated RDW preoperatively may not necessarily be actionable, despite evidence of its association with short term mortality. However, the strength of using RDW as a prognostic biomarker lies with its ready availability at no extra cost, since it is routinely performed as part of a full blood count

### Data Availability

Data from this study are available for download from the Dyrad Digital Repository at the following 10.5061/dryad.5772v.

## Conclusion

Preoperative elevated RDW is an independent predictor of 30-day postoperative mortality among older patients undergoing non-cardiac surgery, and has an incremental effect on preoperative anaemia in predicting this risk. RDW could be used to improve existing risk stratification strategies and further studies are needed to explore this role and the biological process responsible for this association between RDW and mortality.
